# MACC1-AS1 promotes hepatocellular carcinoma cell invasion and proliferation by regulating PAX8

**DOI:** 10.18632/aging.102585

**Published:** 2020-01-08

**Authors:** Hui Tong, Xiaohui Liu, Tao Li, Weihua Qiu, Chenghong Peng, Baiyong Shen, Zhecheng Zhu

**Affiliations:** 1 Department of General Surgery, Ruijin Hospital, Shanghai Jiao Tong University School of Medicine, Shanghai 200025, China; 2 CNRS-LIA124, Sino-French Research Center for Life Sciences and Genomics, State Key Laboratory of Medical Genomics, Ruijin Hospital, Shanghai Jiao Tong University School of Medicine, Shanghai 200025, China

**Keywords:** hepatocellular carcinoma, lncRNAs, MACC1-AS1, PAX8

## Abstract

Long noncoding RNAs play vital roles in several biological processes, including cell growth and embryonic development. We showed that MACC1-AS1 was overexpressed in hepatocellular carcinoma (HCC) cells and tissues. The MACC1-AS1 expression level was dramatically upregulated in HCC samples compared to adjacent normal samples, and 77.5% (31 of 40) of HCC samples showed overexpression of MACC1-AS1. Ectopic MACC1-AS1 expression enhanced cell proliferation and cyclin D1 expression in both SMMC7721 and MHCC-97H cells. Ectopic expression of MACC1-AS1 promoted vimentin, N-cadherin and snail expression and decreased E-cadherin expression in both SMMC7721 and MHCC-97H cells. MACC1-AS1 overexpression also induced cell invasion in the same two cell lines. Furthermore, MACC1-AS1 overexpression enhanced PAX8 expression in HCC cells. The PAX8 level was dramatically increased in HCC samples compared to adjacent normal samples, and 75% (30 of 40) of HCC samples showed overexpression of PAX8. PAX8 expression was positively correlated with MACC1-AS1 expression in HCC samples. MACC1-AS1 overexpression promoted HCC cell proliferation, EMT and invasion through regulating PAX8. These results suggest that MACC1-AS1 acts as an oncogene in the development of HCC.

## INTRODUCTION

Hepatocellular carcinoma (HCC) ranks as the 5^th^ most common tumor worldwide and the 3^rd^ most common cause of tumor-associated death [1–4]. Due to several risk factors, such as hepatitis C and hepatitis B virus infection, alcohol abuse and aflatoxin-contaminated food, HCC incidence has increased in the last 2 decades [5–9]. As an aggressive solid tumor, HCC is characterized by early metastasis, rapid infiltration and proliferation, poor prognosis and high-grade malignancy [10–14]. Despite progression in therapies including surgery, interventional therapy, radiation and chemotherapy, the prognosis of advanced HCC remains unsatisfactory [15–18]. Therefore, it is necessary to find early diagnosis markers and new therapeutic targets for HCC.

Long noncoding RNAs (lncRNAs) are a subgroup of ncRNAs that are more than two hundred nucleotides in length with limited or no protein coding potential [19–23]. Multiple studies have suggested that lncRNAs are involved in several cellular processes, such as cell migration, proliferation, apoptosis, differentiation and invasion [21, 24–26]. Moreover, many lncRNAs were found to be deregulated in several cancers and were correlated with cancer growth and carcinogenesis [27–31]. Recently, a new lncRNA MACC1-AS1 was shown to play critical roles in the development of tumors, such as pancreatic carcinoma and gastric cancer [32–34]. Mesenchymal stem cells (MSCs) secrete TGF-β1 induce MACC1-AS1 expression in gastric cancer cells, which enhances fatty acid oxidation-dependent chemoresistance and stemness by antagonizing miR-145-5p expression [33]. Zhao et al. [34] showed that MACC1-AS1 levels were overexpressed in gastric cancer samples, and overexpression of MACC1-AS1 increased gastric cancer cell growth and suppressed cell apoptosis partly by regulating AMPK/Lin28. Qi et al. [32] demonstrated that MACC1-AS1 levels were upregulated in pancreatic carcinoma samples and that knockdown of MACC1-AS1 suppressed pancreatic carcinoma cell growth and metastasis. However, the role and function of MACC1-AS1 in HCC development remain unknown.

In this research, we studied the expression and function of MACC1-AS1 in HCC development. We found that MACC1-AS1 was overexpressed in HCC cells and tissues. Ectopic MACC1-AS1 expression promoted cell proliferation and cyclin D1 expression in HCC cells.

## RESULTS

### MACC1-AS1 was overexpressed in HCC cells

**Figure 1:**
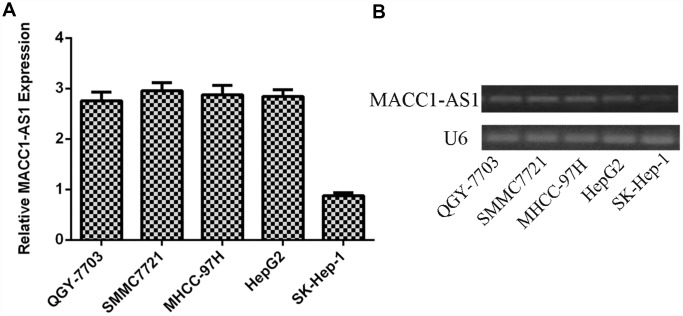
**MACC1-AS1 was overexpressed in HCC cells.** (**A**) The expression of MACC1-AS1 in four HCC cell lines (QGY-7703, SMMC7721, MHCC-97H and HepG2) and one hepatocyte cell line (HL-7702) was detected by qRT-PCR. (**B**) The expression of MACC1-AS1 in four HCC cell lines (QGY-7703, SMMC7721, MHCC-97H and HepG2) and one hepatocyte cell line (HL-7702) was detected by PCR.

We first analyzed MACC1-AS1 expression in HCC cell lines and one normal hepatocyte cell line. As indicated in Figure 1A, MACC1-AS1 was overexpressed in four HCC cell lines (QGY-7703, SMMC7721, MHCC-97H and HepG2) compared to the normal hepatocyte cell line (HL-7702). In line with this, we demonstrated that the MACC1-AS1 was overexpressed in four HCC cell lines (QGY-7703, SMMC7721, MHCC-97H and HepG2) compared to the hepatocyte cell line (HL-7702) by using RT-PCR.

### MACC1-AS1 was overexpressed in HCC samples

**Figure 2:**
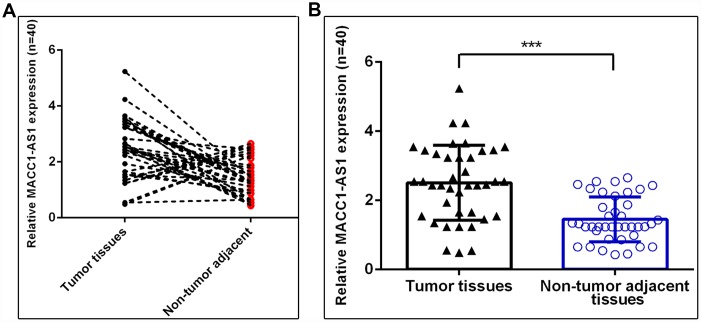
**MACC1-AS1 was overexpressed in HCC samples.** (**A**) MACC1-AS1 level was dramatically increased in HCC samples compared to adjacent normal samples, and 77.5% (31 of 40) of HCC samples showed overexpression of MACC1-AS1. (**B**) MACC1-AS1 expression in 40 paired HCC and adjacent normal samples were analyzed. ***p<0.001.

Furthermore, we analyzed MACC1-AS1 expression in 40 paired HCC and adjacent normal samples. As indicated in Figure 2A, the MACC1-AS1 expression level was dramatically upregulated in HCC samples compared to adjacent normal samples, and 77.5% (31 of 40) of HCC samples showed overexpression of MACC1-AS1. In summary, MACC1-AS1 expression was higher in HCC tissues than in nontumor tissues (Figure 2B).

### Ectopic expression of MACC1-AS1 induced HCC cell growth

**Figure 3:**
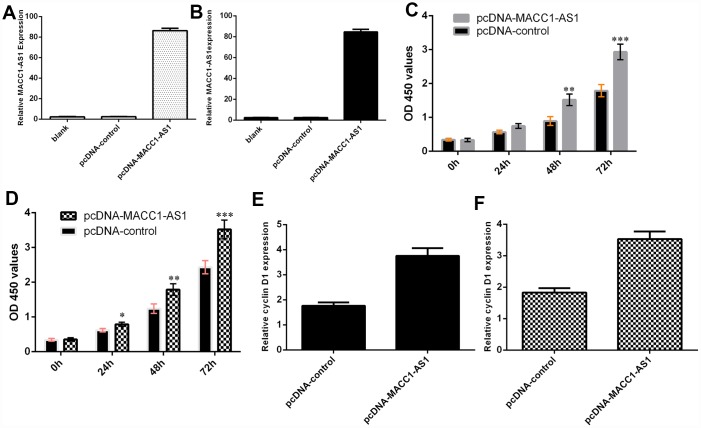
**Ectopic expression of MACC1-AS1 induced HCC cell growth.** (**A**) MACC1-AS1 expression was detected in the SMMC7721 cell by using qRT-PCR analysis. (**B**) MACC1-AS1 expression was detected in the MHCC-97H cell by using qRT-PCR analysis. (**C**) Ectopic MACC1-AS1 expression promoted cell proliferation in SMMC7721 cell. (**D**) Overexpression of MACC1-AS1 induced MHCC-97H cell growth. (**E**) Elevated MACC1-AS1 expression induced the cyclin D1 expression in SMMC7721 cell. (**F**) The expression of cyclin D1 was analyzed by using qRT-PCR. *p<0.05, **p<0.01 and ***p<0.001.

We confirmed that the expression of MACC1-AS1 was significantly upregulated in SMMC7721 cells (Figure 3A) and MHCC-97H cells (Figure 3B) after transfection with the pcDNA-MACC1-AS1 vector. Ectopic MACC1-AS1 expression promoted cell proliferation in both SMMC7721 cells (Figure 3C) and MHCC-97H cells (Figure 3D). We determined by qRT-PCR that elevated MACC1-AS1 expression induced cyclin D1 expression in both SMMC7721 cells (Figure 3E) and MHCC-97H cells (Figure 3F).

### Elevated MACC1-AS1 expression promoted HCC cell epithelial-mesenchymal transition and invasion

**Figure 4:**
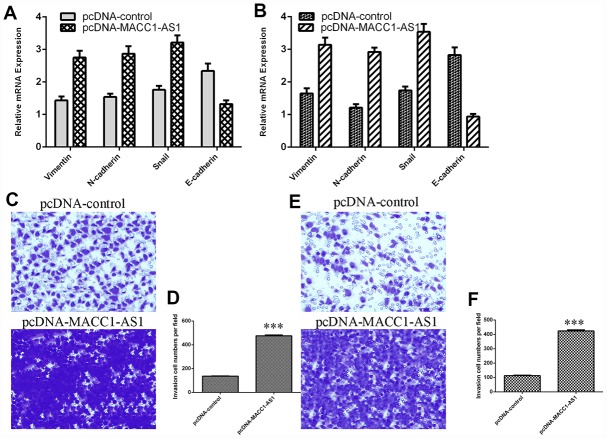
**Elevated MACC1-AS1 expression promoted HCC cell epithelial-mesenchymal transition and invasion.** (**A**) Ectopic MACC1-AS1 expression promoted vimentin, N-cadherin and snail expression and inhibited E-cadherin expression in SMMC7721 cell. (**B**) The mRNA expression of vimentin, N-cadherin, snail and E-cadherin was detected by qRT-PCR. (**C**) MACC1-AS1 overexpression induced the cell invasion in SMMC7721 cell. (**D**) The relative number of invasive cells is shown. (**E**) MACC1-AS1 overexpression induced the cell invasion in MHCC-97H cell. (**F**) The relative number of invasive cells is shown. ***p<0.001.

Ectopic MACC1-AS1 expression promoted vimentin, N-cadherin and snail mRNA expression and inhibited E-cadherin mRNA expression in both SMMC7721 cells (Figure 4A) and MHCC-97H cells (Figure 4B). In addition, MACC1-AS1 overexpression induced cell invasion in both SMMC7721 cells (Figure 4C) and MHCC-97H cells (Figure 4E). The relative number of invasive cells is shown in Figure 4D and Figure 4F.

### MACC1-AS1 overexpression enhanced PAX8 expression in HCC cells

**Figure 5:**
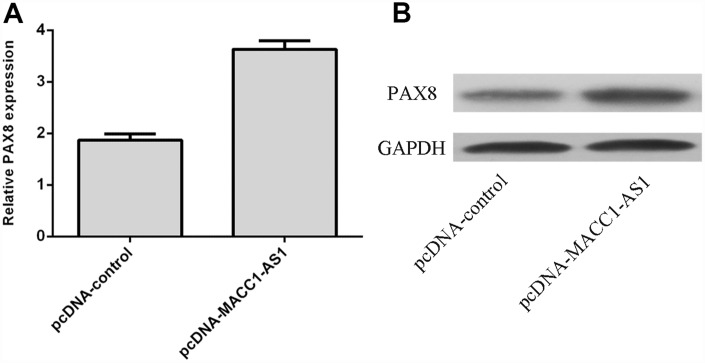
**MACC1-AS1 overexpression enhanced PAX8 expression in HCC cells.** (**A**) Overexpression of MACC1-AS1 enhanced PAX8 expression in SMMC7721 cell, as determined by qRT-PCR. (**B**) Elevated expression of MACC1-AS1 promoted the PAX8 expression in SMMC7721 cell, as determined by western blotting.

Overexpression of MACC1-AS1 enhanced PAX8 expression in SMMC7721 cells, as determined by using qRT-PCR (Figure 5A). Moreover, we demonstrated that elevated expression of MACC1-AS1 promoted PAX8 expression in SMMC7721 cells via western blot analysis (Figure 5B).

### PAX8 expression was upregulated in HCC tissues

**Figure 6:**
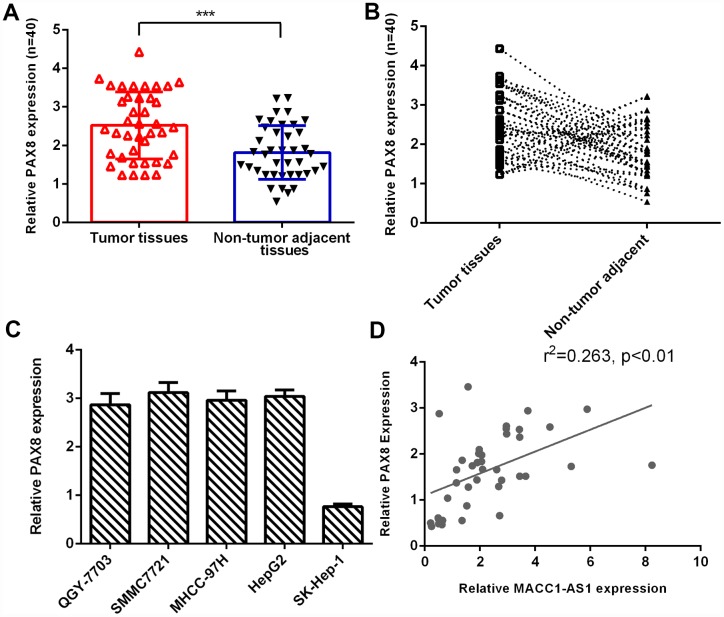
**PAX8 expression was upregulated in HCC tissues.** (**A**) PAX8 expression was higher in HCC tissues than in nontumor tissues. (**B**) Compared to adjacent normal samples, 75% (30 of 40) of HCC samples showed overexpression of PAX8. (**C**) PAX8 was overexpressed in four HCC cell lines (QGY-7703, SMMC7721, MHCC-97H and HepG2) compared to the hepatocyte cell line (HL-7702). (**D**) PAX8 expression was positively correlated with MACC1-AS1 expression in HCC samples. ***p<0.001.

As indicated in Figure 6A, PAX8 expression was higher in HCC tissues than in nontumor tissues. The PAX8 level was dramatically increased in HCC samples compared to adjacent normal samples, and 75% (30 of 40) of HCC samples showed overexpression of PAX8 (Figure 6B). PAX8 was overexpressed in four HCC cell lines (QGY-7703, SMMC7721, MHCC-97H and HepG2) compared to the hepatocyte cell line (HL-7702) (Figure 6C). Moreover, we indicated that PAX8 expression was positively correlated with MACC1-AS1 expression in HCC samples (Figure 6D).

### MACC1-AS1 overexpression promoted HCC cell proliferation, EMT and invasion through regulating PAX8

**Figure 7:**
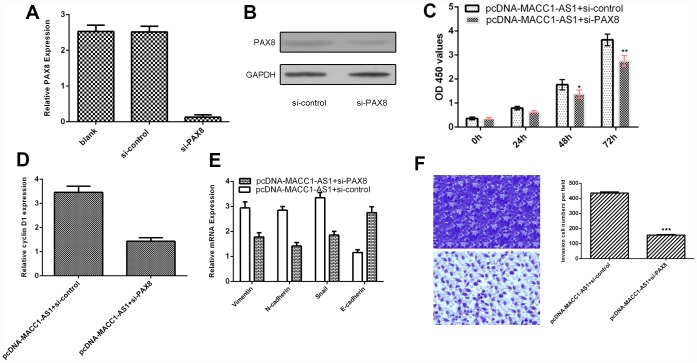
**MACC1-AS1 overexpression promoted HCC cell proliferation, EMT and invasion through regulating PAX8.** (**A**) The expression of PAX8 was detected in SMMC7721 cell by using qRT-PCR assay. (**B**) The protein expression of PAX8 was analyzed by using western blotting. (**C**) Knockdown expression of PAX8 suppressed cell proliferation in MACC1-AS1-overexpressing SMMC7721 cells. (**D**) Inhibition of PAX8 expression decreased cyclin D1 expression in MACC1-AS1-overexpressing SMMC7721 cells. (**E**) Knockdown PAX8 expression inhibited vimentin, N-cadherin and snail expression and promoted E-cadherin expression in MACC1-AS1-overexpressing SMMC7721 cell. (**F**) Knockdown expression of PAX8 decreased cell invasion MACC1-AS1-overexpressing SMMC7721 cell. The relative invasive cells were shown in the right. *p<0.05, **p<0.01 and ***p<0.001.

To further study the role of PAX8 in MACC1-AS1 modulation cell function, we transfected siRNA-PAX8 into MACC1-AS1-overexpressing SMMC7721 cells. We demonstrated that the expression of PAX8 was downregulated in SMMC7721 cells after transfection with si-PAX8, as determined by qRT-PCR (Figure 7A). In addition, we found that PAX8 levels were decreased in SMMC7721 cells after transfection with si-PAX8 by using western blotting (Figure 7B). Knockdown of PAX8 suppressed cell proliferation in MACC1-AS1-overexpressing SMMC7721 cells (Figure 7C). Inhibited expression of PAX8 decreased cyclin D1 expression in MACC1-AS1-overexpressing SMMC7721 cells (Figure 7D). Knockdown of PAX8 expression inhibited vimentin, N-cadherin and snail expression and promoted E-cadherin expression in MACC1-AS1-overexpressing SMMC7721 cells (Figure 7E). Knockdown of PAX8 decreased the invasion of MACC1-AS1-overexpressing SMMC7721 cells (Figure 7F).

## DISCUSSION

In this study, MACC1-AS1 was overexpressed in HCC cells and tissues. The MACC1-AS1 level was dramatically increased in HCC samples compared to adjacent normal samples, and 77.5% (31 of 40) of HCC samples showed overexpression of MACC1-AS1. Ectopic expression of MACC1-AS1 promoted cell proliferation and cyclin D1 expression in both SMMC7721 and MHCC-97H cells. Ectopic expression of MACC1-AS1 promoted vimentin, N-cadherin and snail expression and inhibited E-cadherin expression in both SMMC7721 and MHCC-97H cells. MACC1-AS1 overexpression induced cell invasion in both SMMC7721 and MHCC-97H cells. Furthermore, MACC1-AS1 overexpression enhanced PAX8 expression in HCC cells. The PAX8 level was dramatically increased in HCC samples compared to adjacent normal samples, and 75% (30 of 40) of HCC samples showed overexpression of PAX8. PAX8 expression was positively correlated with MACC1-AS1 expression in HCC samples. MACC1-AS1 overexpression promoted HCC cell proliferation, EMT and invasion through regulating PAX8.

Extensive research has indicated that lncRNA MACC1-AS1 plays critical roles in the development of tumors such as pancreatic carcinoma and gastric cancer [32–34]. Qi et al [32]. showed that MACC1-AS1 was overexpressed in pancreatic carcinoma tissues and that knockdown of MACC1-AS1 inhibited pancreatic carcinoma cell proliferation and metastasis. He and his colleagues indicated that MSCs secrete TGF-β1 and induce the expression of MACC1-AS1 in gastric tumor cells, which promotes fatty acid oxidation-dependent chemoresistance and stemness by antagonizing miR-145-5p expression [33]. Zhao et al [34]. demonstrated that MACC1-AS1 expression was upregulated in gastric cancer tissues and that overexpression of MACC1-AS1 induced gastric cancer cell growth and suppressed cell apoptosis partly via regulating AMPK/Lin28. However, the role and function of MACC1-AS1 in HCC development remain unknown. Therefore, we first detected MACC1-AS1 expression in HCC cells. We showed that MACC1-AS1 was overexpressed in four HCC cell lines (QGY-7703, SMMC7721, MHCC-97H and HepG2) compared to the hepatocyte cell line (HL-7702). We analyzed MACC1-AS1 expression in 40 paired HCC and adjacent normal samples. The MACC1-AS1 level was dramatically increased in HCC samples compared to adjacent normal samples, and 77.5% (31 of 40) of HCC samples showed overexpression of MACC1-AS1. Ectopic expression of MACC1-AS1 promoted cell proliferation and cyclin D1 expression in both SMMC7721 and MHCC-97H cells. Ectopic expression of MACC1-AS1 promoted vimentin, N-cadherin and snail expression and inhibited E-cadherin expression in both SMMC7721 and MHCC-97H cells. MACC1-AS1 overexpression induced cell invasion in both SMMC7721 and MHCC-97H cells.

Increasing evidence indicates that PAX8 plays important roles in tumor development [35–37]. For example, Bie et al [36]. showed that PAX8 was overexpressed in gastric tumors, and knockdown of PAX8 inhibited cell cycle progression, colony formation and proliferation. Ghannam-Shahbari et al. indicated that PAX8 played an antiapoptotic and pro-proliferative role in high-grade serous carcinoma. Chai et al. [38] demonstrated that PAX8 was overexpressed in ovarian cancer and that high expression of PAX8 was correlated with FIGO stage, survival rate and cell differentiation degree. Furthermore, Wang et al. demonstrated that silenced expression of PAX8 suppressed hepatoma cell clonogenicity and proliferation and growth in vivo [39]. However, the expression of PAX8 in HCC tissues remains unknown. In this study, we showed that the PAX8 level was dramatically increased in HCC samples compared to adjacent normal samples, and 75% (30 of 40) of HCC samples showed overexpression of PAX8. PAX8 was overexpressed in four HCC cell lines (QGY-7703, SMMC7721, MHCC-97H and HepG2) compared to the hepatocyte cell line (HL-7702). In addition, MACC1-AS1 overexpression enhanced PAX8 expression in HCC cells. PAX8 expression was positively correlated with MACC1-AS1 expression in HCC samples. Furthermore, we indicated that MACC1-AS1 overexpression promoted HCC cell proliferation, EMT and invasion by regulating PAX8.

In conclusion, we showed that the MACC1-AS1 level is upregulated in HCC cell lines and tissues. MACC1-AS1 overexpression promoted HCC cell proliferation, EMT and invasion through regulating PAX8. These results suggest that MACC1-AS1 acts as an oncogene in the development of HCC.

## MATERIALS AND METHODS

### Tissues

Our research was performed with written consent from each patient. This study followed the Declaration of Helsinki and was approved by Ruijin Hospital, Shanghai Jiao Tong University School of Medicine. Forty pairs of HCC tissues and adjacent normal tissues were collected from Ruijin Hospital, Shanghai Jiao Tong University School of Medicine. These tissues were stored in liquid nitrogen until use.

### Cell culture and transfection

Four HCC cell lines (QGY-7703, SMMC7721, MHCC-97H and HepG2) and a hepatocyte cell line (HL-7702) were obtained from ATCC. Cell lines were kept in DMEM supplemented with FBS and antibiotic. siRNA-control, siRNA-PAX8 and pcDNA-MACC1-AS1 and pcDNA-control were obtained from Genepharma and then transfected into HCC cells by using Lipofectamine 3000 according to the manufacturer’s instructions.

### RNA extraction and real-time quantitative PCR (qRT-PCR)

RNA was extracted from cells and tissues by using TRIzol (Invitrogen, CA, USA). The mRNA expression was quantified by performing a qRT-PCR assay on the iQ5 PCR System (Bio-Rad, USA) using SYBR Green. GAPDH was used as a normalization control. Data were analyzed by the 2^-ΔΔCt^ method.

### Cell growth and invasion assays

Cell growth was evaluated using CCK-8 (Dojindo, Japan) following the manufacturer’s instructions. Cells were cultured in 96 well plates and detected 0, 24, 48 and 72 hours after transfection. The optical absorbance was read at 450 nm. Cell invasion was evaluated in a transwell chamber. Cells were plated in the upper compartment with serum-free medium, and FBS was added to the lower chamber. After 48 hours, the cells were fixed, stained and then counted.

### Western blotting

Total sample or cellular protein was extracted by using lysis buffer, and the concentration of protein was measured by using a BCA kit (Pierce, Rockford, IL). Total protein was separated by 10% SDS-PAGE and transferred to the nitrocellulose membrane. After incubating in 5% nonfat milk, the membrane was probed with primary antibodies (anti-PAX8 and anti-GAPDH) at 4°C overnight. The membrane was washed three times in TBST and then probed with horseradish peroxidase-conjugated secondary antibody. The protein signal was determined with an enhanced chemiluminescent reagent (Millipore, USA).

### Statistical analysis

SPSS version 18 (Chicago, USA) was applied to statically analyze the data. Data are indicated as the mean ± SD. Differences between these groups were detected using Student’s t-test. A P value < 0.05 was deemed statistically significant.
